# Identification of Cbp1, a c-di-GMP Binding Chemoreceptor in *Azorhizobium caulinodans* ORS571 Involved in Chemotaxis and Nodulation of the Host Plant

**DOI:** 10.3389/fmicb.2019.00638

**Published:** 2019-04-02

**Authors:** Yu Sun, Zhihong Xie, Fu Sui, Xiaolin Liu, Wuzeng Cheng

**Affiliations:** ^1^Key Laboratory of Coastal Biology and Bioresource Utilization, Yantai Institute of Coastal Zone Research, Chinese Academy of Sciences, Yantai, China; ^2^College of Resources and Environment, University of Chinese Academy of Sciences, Beijing, China; ^3^Center for Ocean Mega-Science, Chinese Academy of Sciences, Qingdao, China; ^4^Shandong Huibang Bohai Agriculture Development Limited Company, Dongying, China

**Keywords:** c-di-GMP, PilZ domain, chemoreceptor, chemotaxis, nodulation

## Abstract

Cbp1, a chemoreceptor containing a PilZ domain was identified in *Azorhizobium caulinodans* ORS571, a nitrogen-fixing free-living soil bacterium that induces nodule formation in both the roots and stems of the host legume *Sesbania rostrata*. Chemoreceptors are responsible for sensing signals in the chemotaxis pathway, which guides motile bacteria to beneficial niches and plays an important role in the establishment of rhizobia-legume symbiosis. PilZ domain proteins are known to bind the second messenger c-di-GMP, an important regulator of motility, biofilm formation and virulence. Cbp1 was shown to bind c-di-GMP through the conserved RxxxR motif of its PilZ domain. A mutant strain carrying a *cbp1* deletion was impaired in chemotaxis, a feature that could be restored by genetic complementation. Compared with the wild type strain, the Δ*cbp1* mutant displayed enhanced aggregation and biofilm formation. The Δ*cbp1* mutant induced functional nodules when inoculated individually. However, the Δ*cbp1* mutant was less competitive than the wild type in competitive root colonization and nodulation. These data are in agreement with the hypothesis that the c-di-GMP binding chemoreceptor Cbp1 in *A. caulinodans* is involved in chemotaxis and nodulation.

## Introduction

C-di-GMP (bis-(3′–5′)-cyclic dimeric guanosine monophosphate) is a second messenger commonly found in the bacterial kingdom that regulates multiple cellular functions including motility, extracellular polysaccharide production and biofilm formation (Hengge, 2009). Generally, high intracellular c-di-GMP concentrations down regulate motility and promote surface attachment, which correlates with the transition from a motile to sessile lifestyle (Povolotsky and Hengge, 2012; Purcell et al., 2012; Roy et al., 2012). C-di-GMP turnover is controlled by diguanylate cyclases (DGCs) and c-di-GMP specific phosphodiesterases (PDEs) (Chan et al., 2004; Rao et al., 2008). C-di-GMP is synthesized from two molecules of GTP by DGCs containing a GGDEF domain with a conserved GG(D/E)EF motif and hydrolyzed into pGpG by PDEs containing an EAL domain or into two molecules of GMP by PDEs containing a HD-GYP domain (Christen et al., 2005; Ryjenkov et al., 2005; Ryan et al., 2006).

C-di-GMP exerts its biological effects by binding to various effectors including riboswitches and proteins (Lee et al., 2007; Newell et al., 2009; Bordeleau et al., 2015). Among these, proteins with PilZ domains have been extensively studied (Ryan et al., 2012). The PilZ domain is characterized by conserved RxxxR and D/NxSxxG motifs responsible for c-di-GMP binding (Benach et al., 2007). PilZ domain proteins display a broad range of c-di-GMP binding affinities and are involved in the control of different bacterial behaviors. In *Escherichia coli*, the PilZ domain protein YcgR inhibits motility at high c-di-GMP concentrations by interacting with the flagellar proteins FliG and FliM (Paul et al., 2010). In *Pseudomonas aeruginosa*, the PilZ domain protein HapZ regulates a two-component signaling pathway by interacting with the histidine kinase SagS (Xu et al., 2016a), while another PilZ domain protein Alg44 is required for alginate biosynthesis (Merighi et al., 2007). In *Xanthomonas oryzae* pv. *oryzae*, PilZ domain proteins were shown to regulate virulence (Yang et al., 2015).

Chemotaxis enables motile bacteria to detect environmental changes, such as chemical gradients, and respond by moving to beneficial niches that support optimal growth. Chemoreceptors (also known as methyl-accepting chemotaxis proteins, MCPs) are signaling proteins that transduce chemotactic signals via interaction with both CheA (histidine kinase) and CheW (coupling protein) of the chemotaxis pathway (Wadhams and Armitage, 2004). As a histidine kinase, CheA can be autophosphorylation with ATP, and CheA-P phosphorylates its response regulator CheY. CheY-P then binds to the flagellar rotary motor to bring about a reversal in rotational direction (Tindall et al., 2012). Chemoreceptor binding of a chemotactic signal molecule regulates the kinase activity of CheA, thus controls the probability of directional changes (Lai et al., 2006). The signal ligand binding affinity of chemoreceptors depends on their methylation state (Li and Weis, 2000). There are chemotaxis adaptation proteins CheB (methylesterase) and CheR (methyltransferase) present in the chemotaxis pathway. CheB is also phosphorylated by CheA-P, and CheB-P competes with CheR to regulate the methylation state of chemoreceptors, thus resetting their signal sensitivity (Wuichet et al., 2007). *Azorhizobium caulinodans* ORS571 is a versatile nitrogen fixer that can fix nitrogen both in the symbiotic and free-living states under microaerobic conditions. It is a symbiont of the legume *Sesbania rostrata* and can form nitrogen fixing nodules both on roots and stems (Dreyfus et al., 1988). Chemotaxis of *A. caulinodans* ORS571 is controlled by a single chemotaxis system that contains five chemotaxis proteins: CheA, CheW, CheY_1_, CheB, and CheR (Liu et al., 2018). Chemotaxis toward chemoattractants in root exudates promotes attachment between rhizobia and legume roots and subsequent infection, thus benefiting the establishment of rhizobia-legume symbiosis (Greer-Phillips et al., 2004; Scharf et al., 2016).

A considerable number of c-di-GMP-related genes were identified in rhizobia (Gao et al., 2014). The c-di-GMP-related genes have been investigated in *Sinorhizobium meliloti* and a single PilZ domain protein, McrA, was identified as a c-di-GMP binding effector able to repress motility at elevated c-di-GMP concentrations (Schäper et al., 2016). The PilZ domain containing chemoreceptor Tlp1 from *Azospirillum brasiencse* has been shown to be involved in the chemotactic signaling pathway (Russell et al., 2013). The integration of c-di-GMP into the chemotactic signaling pathway prompted us to investigate the potential interplay between c-di-GMP and chemotaxis in *A. caulinodans* ORS571. The whole genome of ORS571 has been sequenced (Lee et al., 2008), and there are 43 chemoreceptors as shown in the Pfam database (Jiang et al., 2016). In the present study, we report the characterization of a PilZ domain containing chemoreceptor that we named Cbp1 (c-di-GMP binding protein 1) and show the involvement of Cbp1 in chemotaxis and symbiosis with legume host *S. rostrata*.

## Materials and Methods

### Bacterial Strains, Plasmids, and Culture Conditions

Strains and plasmids used in this study are listed in Table 1. *A. caulinodans* ORS571 and mutant strain derivatives were grown at 37°C in TY medium and L3 minimal medium with the corresponding antibiotics (Jiang et al., 2016). *E. coli* strains used for cloning and expression were routinely grown at 37°C in LB medium. Solid media plates contained 1.5% agar. Antibiotics were used at the following concentrations: ampicillin 100 μg/mL, nalidixic acid 25 μg/mL, kanamycin 25 μg/mL, gentamycin 50 μg/mL, and tetracycline 10 μg/mL.

**Table 1:** Strains and plasmids used in this study.

Strain or plasmid	Relevant characteristics^a^	Source or reference
**Strains**		
***E. coli***		
Top10	F^-^ *mcrA* Δ(*mrr-hsd*RMS*-mcr*BC) φ80 *lac*ZΔM15Δ *lac*X74 *rec*A1 *ara*Δ139Δ(*ara-leu*)7697 *gal*U *gal*K *rps*L (Str^R^) *end*A1 *nup*G	Transgen
***Azorhizobium caulinodans***		
ORS571	Type strain; Amp^R^, Nal^R^	Nakajima et al., 2012
Δ*cbp1*	ORS571 derivative; deletion of the *cbp1* gene, Amp^R^, Nal^R^	This study
**Plasmids**		
pCM351	Recombinant vector for gentamycin substitution, *cre/loxp*, Gen^R^,Tc^R^	Marx and Lidstrom, 2002
pCM157	IncP plasmid that expresses Cre recombinase; Tc^R^	Marx and Lidstrom, 2002
pRK2013	Helper plasmid, ColE1 replicon; Tra^+^ Km^R^	Figurski and Helinski, 1979
pBBR1MCS-2 pBBR-*cbp1*	Broad host range plasmid; Km^R^ pBBR1MCS-2 with *cbp1* gene and promoter region; Km^R^	Kovach et al., 1995 This study
pBBR-*cbp1_R320A_*	pBBR-*cbp1* containing R320A mutantion; Km^R^	This study
pBAD/M	General expression vector, C-His6 tag; Amp^R^	Sun et al., 2015
pBAD/M-*cbp1*	pBAD/M with ORF of *cbp*1 gene; Amp^R^	This study
pBAD/M-*cbp1_R320A_*	pBAD/M with *cbp1* gene containing R320A mutation; Amp^R^	This study

*Amp^*R*^, ampicillin resistance; Nal^*R*^, Nalidixic acid resistance; Gen^*R*^, gentamicin resistance; Tc^*R*^, tetracycline resistance; Km^*R*^, kanamycin resistance.*

### Protein Expression and Purification

Plasmid pBAD/M with a C-terminal 6×His tag was used to overexpress Cbp1 in *E. coli* Top10 (Sun et al., 2015). A DNA fragment encoding Cbp1 was amplified from *A. caulinodans* ORS571 genomic DNA with the primer pair Cbp1-*Nde*I-F/*Hin*dIII-R and cloned into the *Nde*I/*Hin*dIII site of pBAD/M to create the recombinant expression plasmid. The resulting plasmid was then transformed into *E. coli* Top10. The Cbp1R320A mutant was generated using the QuikChange^®^Site-Directed Mutagenesis Kit (Stratagene) with the primer pair Cbp1R320A-F/R. Protein expression was induced with 0.1% L-arabinose and incubation at 16°C for 24 h. Protein purification was performed as described previously (Sun et al., 2015). Proteins were analyzed by SDS-PAGE and their concentration was measured using the standard BCA protein assay.

### Protein Thermal Shift Assay

The protein thermal shift assay was performed as described previously with some modifications (Huynh and Partch, 2015). The StepOne Plus Quantitative Real-time PCR system (Thermo Fisher Scientific) was used to determine the thermal shift of purified proteins in the presence of SYPRO orange dye, which binds hydrophobic regions of proteins. The reaction buffer was 25 mM Tris–HCl (pH7.9) with 10 mM MgCl_2_ and the final assay concentration of SYPRO orange dye was 1× (stock solution was 5000×). For measurement of protein thermal shift without a ligand, 5 μM of purified Cbp1 or Cbp1R320A protein was added. For measurement of protein thermal shift with a ligand, 5 μM of the purified protein was added with 5 μM c-di-GMP. 50 μL of reaction buffer with 1 × SYPRO orange was used as a negative control (No Protein Control, NPC). Before being measured in the qRT-PCR instrument, all reaction mixtures were incubated for 1 h at 37°C. The experimental parameters were selected as described by Huynh and Partch (2015). The experimental data was analyzed using Protein Thermal Shift Software 1.3 (Thermo Fisher Scientific).

### Isothermal Titration Calorimetry (ITC)

Isothermal titration calorimetry measurements were performed as described previously with some modifications (Huang et al., 2016). A NANO ITC 2G (TA Instruments) was used to detect c-di-GMP binding to Cbp1 and Cbp1R320A. Both the Cbp1 and Cbp1R320A proteins and the ligand c-di-GMP were dissolved in buffer (25 mM Tris–HCl, 10 mM MgCl_2_, pH7.9). 20 μM of the purified protein was titrated with aliquots of 300 μM c-di-GMP. The experimental data was analyzed using NanoAnalyze software 3.5.0 (TA Instruments).

### Construction of Mutant and Complemented Strains

The Δ*cbp1* mutant was constructed using the allelic exchange vectors pCM351 and pCM157, a pair of vectors carrying the *cre/lox* system (Marx and Lidstrom, 2002). 702-bp upstream and 690-bp downstream fragments of the *cbp1* gene were amplified from *A. caulinodans* ORS571 genomic DNA with the primer pairs Cbp1Up-*Xba*I-F/*Nde*I-R and Cbp1Down-*Age*I-F/*Sac*I-R. The fragments were then digested and inserted into the plasmid vector pCM351. The resulting recombinant plasmid (pCM351:Up:Down) was introduced into *A. caulinodans* ORS571 by tri-parental conjugation with the helper plasmid pRK2013 (Figurski and Helinski, 1979). Allelic exchange between the recombinant plasmid pCM351:Up:Down and the ORS571 chromosome led to the replacement of *cbp1* with a gentamicin gene cassette and the cassette was further deleted following the introduction of plasmid pCM157. To complement the Δ*cbp1* mutant, full-length *cbp1* and 300-bp upstream encompassing the predicted promoter region was amplified by PCR using the primer pair Cbp1P-*Kpn*I-F/*Sac*I-R. The amplified fragment was then cloned into the *Kpn*I/*Sac*I site of the broad-host-range vector pBBR1MCS-2 (Kovach et al., 1995) to create the plasmid pBBR-*cbp1*. The plasmid pBBR-*cbp1_R320A_* was then constructed using the site-directed mutagenesis protocol mentioned above with primer pair Cbp1R320A-F/R. The complementation plasmids were introduced into the Δ*cbp1* mutant by tri-parental conjugation with the helper plasmid pRK2013. The empty plasmid pBBR1MCS-2 was also introduced into wild type ORS571 and Δ*cbp1* mutant as control. All primers used in mutant construction are listed in Table 2.

**Table 2:** Primers used in this study.

Primers	Sequences (5′–3′)
Cbp1Up-*Xba*I-F	CGTCTAGACGCGTGCTCGACCTC
Cbp1Up-*Nde*I-F	CGCATATGCGTTCCTCCGGGCA
Cbp1Down-*Age*I-F	CGACCGGTGGCTATTGCCGCTC
Cbp1Down-*Sac*I-R	CGAGCTCGGCACGGCCATATC
Cbp1P-*Kpn*I-F	CGGGGTACCAGTACATGCCCTGAC
Cbp1P-*Sac*I-R	CGAGCTCTACATCCGGTAGCAGGT
Cbp1-*Nde*I-F	CGCATATGTTTGGTTTGTGGG
Cbp1-*Hin*dIII-R	CCAAGCTTGATCGGCGTGCTGAACA
Cbp1R320A-F	GGCGACCGCGCCAAGTTCGAC
Cbp1R320A-R	GTCGAACTTGGCGCGGTCGCC

### Growth Curve Assay

Growth of the strains were measured as previously described (Liu et al., 2017). ORS571 and the mutants were grown overnight in TY medium at 37°C. The overnight-grown cultures were collected by centrifugation and washed three times with L3+N liquid medium. The cells were then inoculated into 50 mL TY medium or L3+N medium (with succinate as a carbon source) at an initial concentration of OD_600_ 0.02 in sterile 250 mL conical flasks. Cells were grown at 150 rpm and 37°C and cell density was monitored every 2 h (0–12 h), 3 h (12–24 h), and 6 h (24–48 h).

### Competitive Capillary Chemotaxis Assay

The competitive capillary chemotaxis assay was performed as previously described with modifications (Liu et al., 2018). Overnight grown cells of ORS571 and mutants were collected by centrifugation, washed three times with PBS buffer, and the suspensions were then adjusted with a final concentration of OD_600_ 0.01. The wild type and each mutant were then mixed at a 1:1 ratio. The 1:1 ratio was re-confirmed by counting CFUs of each strain through serial dilution. 200 μL aliquots of the mixed cell suspensions were placed into the wells of a 96-well plate. Capillaries were heat-sealed at one end using a flame and the open end was then inserted into the PBS buffer and 10 mM succinate solution for 5 min. Solution-filled capillaries were then removed to wells containing bacterial mixtures. The 96-well plates were incubated at 37°C for 1 h. The sealed ends of then capillaries were broken and the contents were then emptied into 1 mL PBS buffer using a rubber adaptor. The ratio of wild type to mutant cells was calculated by counting CFUs of each strain entering the capillaries.

### Aggregation Assay

Overnight grown cells of ORS571 and the Δ*cbp1* mutant were collected by centrifugation and washed three times with L3+N liquid medium. Cells were then inoculated into 15 mL fresh L3+N medium (with succinate as a carbon source) to an initial concentration of OD_600_ 0.02 in sterile 50 mL conical centrifuge tubes. The tubes were then incubated in an orbital shaker and shaken at 220 rpm for 24 and 48 h at 37°C. The tubes were taken out and left standing for a further 20 min. When the aggregated cells settled to the bottom of the tubes, the OD_600_ of the upper cell suspension was measured. The aggregated cells were then vortexed and the total OD_600_ of the cell cultures was measured. The aggregation ratio of each strain was then calculated using the formula: aggregation ratio % = 100^∗^(OD_total_-OD_suspension_)/OD_total_.

### Quantification of Biofilm Formation

Overnight grown cells of wild type and the Δ*cbp1* mutant were collected by centrifugation and washed with L3+N liquid medium. 1.5 mL of resuspended cells with a final concentration of OD_600_ 0.1 was added to a sterile glass tube. The strains were then incubated at 37°C for 3 days. For crystal violet staining, the cells were removed and the glass tubes were washed carefully with sterile water. 2 mL of 1% crystal violet was added to each tube. After 20 min of staining, crystal violet was removed and 1 mL of 30% acetic acid was added to dissolve the crystal violet-stained biofilms. The OD_595_ of solution from each tube was then measured for biofilm quantification.

### Plant Growth, Root Colonization and Nodule Occupancy Competition Assays

*Sesbania rostrata* seeds were treated with concentrated sulfuric acid for 30 min and washed thoroughly with sterile water and then soaked in sterile water in the dark at 37°C for 48 h, as previously described (Jiang et al., 2016). For the root colonization assay, overnight grown cells of wild type and the Δ*cbp1* mutant were harvested and resuspended in sterile L3-N liquid medium. Normalized cell suspensions of wild type and the Δ*cbp1* mutant were mixed in a 1:1 ratio and added into sterile vermiculite. The 1:1 ratio was re-confirmed by counting CFUs of each strain. The germinated seedlings were then transferred into vermiculite for 4 and 24 h. After that, the seedlings were removed, washed and then mashed to recover bacteria from the root surface. Serial dilutions were then plated on TY plates. After growth at 37°C, at least 200 single colonies were analyzed by PCR with the primer pair Cbp1Up-*Xba*I-F /Cbp1Down-*Sac*I-R to calculate the colonization occupation ratio. For nodulation analysis, germinated seedlings were planted in vermiculite in Leonard jars. Nodulation on roots was performed by adding cell cultures into vermiculite, and nodulation on stems was performed by inoculation of stem-root primordia with sterile cotton wool moistened with a bacterial suspension as previously described (Liu et al., 2018). For individual nodulation, cells of a single strain were used. For competitive nodulation, cells of wild type and the Δ*cbp1* mutant were mixed in a 1:1 ratio (re-confirmed by counting CFUs of each strain). All plants were grown at 26°C with a daylight illumination period of 12 h in a greenhouse. The root and stem nodules were harvested 4 weeks after inoculation. Bacteria were then re-isolated from crushed nodules and colonies were tested by PCR as above to determine the nodulation occupation ratio.

### Acetylene Reduction Activity (ARA) Assays

Acetylene reduction activity of the nodules was determined using gas chromatography (Agilent Technologies, 7890A). The ARA was expressed as μmol of C_2_H_4_ production/h/g of fresh nodule, as described by Liu et al. (2017). Each measurement was repeated at least three times.

## Results

### The Chemoreceptor Cbp1 Binds c-di-GMP Through the Conserved RxxxR Motif

Of the 43 chemoreceptors in *A. caulinodans* ORS571 (Jiang et al., 2016), the only chemoreceptor containing a PilZ domain is encoded by the gene *AZC_3349*, here named Cbp1. Cbp1 is an internal soluble protein without a transmembrane region consisting of an N-terminal MA domain (methyl-accepting chemotaxis-like domain) and a C-terminal PilZ domain (Supplementary Figure S1A). All chemoreceptors were identified by the presence of a MA domain, a highly conserved cytoplasmic signaling domain that interacts with the CheA histidine kinase and CheW coupling protein (Wadhams and Armitage, 2004).

**FIGURE 1:**
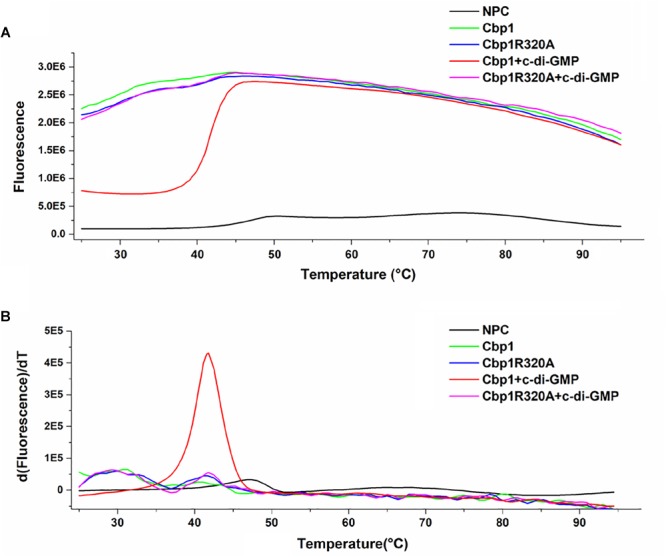
Protein thermal shift assay of Cbp1 and Cbp1R320A. **(A)** The thermal denaturation profile of proteins. **(B)** The profile of the derivative of the fluorescence emission as a function of temperature (dF/dT). NPC (no protein control, black line), Cbp1 (green line), Cbp1R320A (blue line), Cbp1 with c-di-GMP (red line), Cbp1R320A with c-di-GMP (magenta line).

**FIGURE 2:**
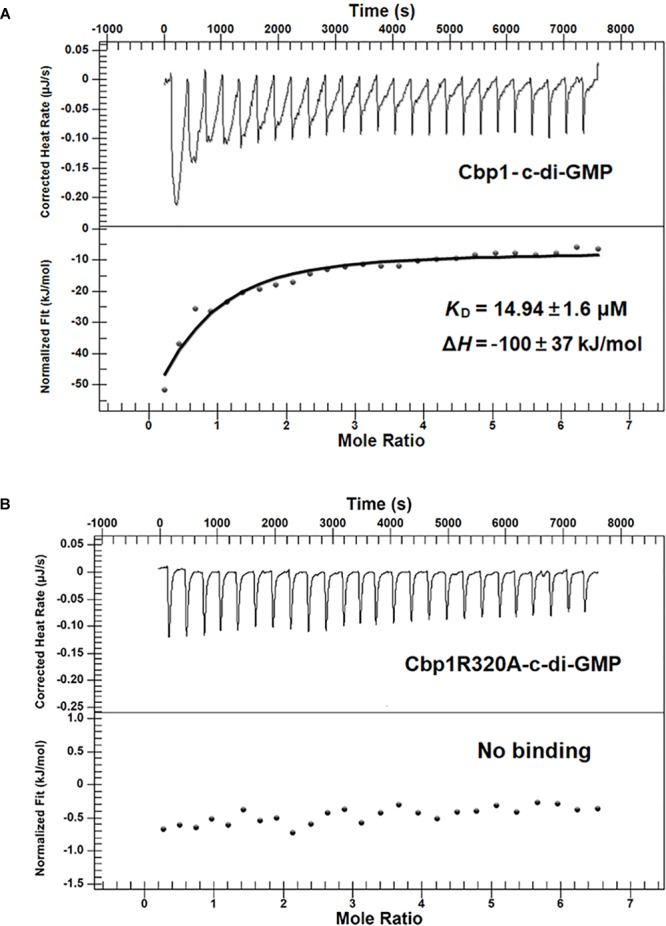
Measurement of the c-di-GMP binding affinity of Cbp1 and Cbp1R320A by ITC. For titration, 20 μM of the purified proteins (Cbp1 and Cbp1R320A) and 300 μM c-di-GMP were used. **(A)** Cbp1 binds c-di-GMP with a *K*_D_ of 14.94 ± 1.6 μM, and the Δ*H* is –100 ± 37 KJ/mol. **(B)** Cbp1R320A cannot bind c-di-GMP.

Multiple alignment of Cbp1 PilZ domain with other proteins showed that the characteristic c-di-GMP binding motifs RxxxR and D/NxSxxG are fully conserved in the Cpb1 PilZ domain (Supplementary Figure S1C). Conservation of the binding motif suggested that Cbp1 may bind c-di-GMP. To determine whether Cbp1 can bind c-di-GMP, Cbp1 was heterologously expressed in *E. coli* Top10 to obtain purified protein (Supplementary Figure S1B). Protein thermal shift assays (also known as different scanning fluorimetry or ThermoFluor) have been widely used to identify stabilizing solution conditions and small-molecule ligands for purified proteins (Huynh and Partch, 2015). The protein thermal shift assay was applied here to analyze the c-di-GMP binding ability of Cbp1. As shown in Figure 1, the denaturation profile of Cbp1 (green line) indicated that Cbp1 had exposed hydrophobic regions in the native state (SYPRO orange interacts with hydrophobic regions in proteins). The red line (Cbp1 with c-di-GMP) indicates a denaturation profile of a well-folded protein: low fluorescence emission at room temperature and then emission increases with increasing temperature. The difference between these lines suggested that Cbp1 could specifically bind c-di-GMP and c-di-GMP binding led to a significant conformational change in Cbp1. In order to better characterize the binding of c-di-GMP to Cbp1, isothermal titration calorimetry (ITC) was performed. Purified Cbp1 was titrated with c-di-GMP and the result of ITC showed that Cbp1 binds c-di-GMP with a *K*_D_ of 14.94 ± 1.6 μM (Figure 2A). A previous study on *A. brasilense* Tlp1 has shown that mutation of Arg563, the first R in RxxxR motif of the PilZ domain, led to complete loss of c-di-GMP binding ability of Tlp1 (Russell et al., 2013). In Cbp1, the corresponding Arg residue is at position 320. Thus, the Arg320 residue of Cbp1 PilZ domain was replaced by Ala. The Cbp1R320A variant was then analyzed for c-di-GMP binding. As expected, there was no obvious change in the denaturation profile of Cbp1R320A with c-di-GMP, which suggested that Cbp1R320A failed to bind c-di-GMP (Figure 1). The ITC analysis of Cbp1R320A further confirmed that it cannot bind c-di-GMP (Figure 2B). Together, the results showed that Cbp1 is a c-di-GMP binding chemoreceptor and the PilZ domain is responsible for c-di-GMP binding.

### The Δ*cbp1* Mutant Is Impaired in Chemotaxis and c-di-GMP Binding Ability of Cbp1 Is Essential for Normal Chemotaxis

**FIGURE 3:**
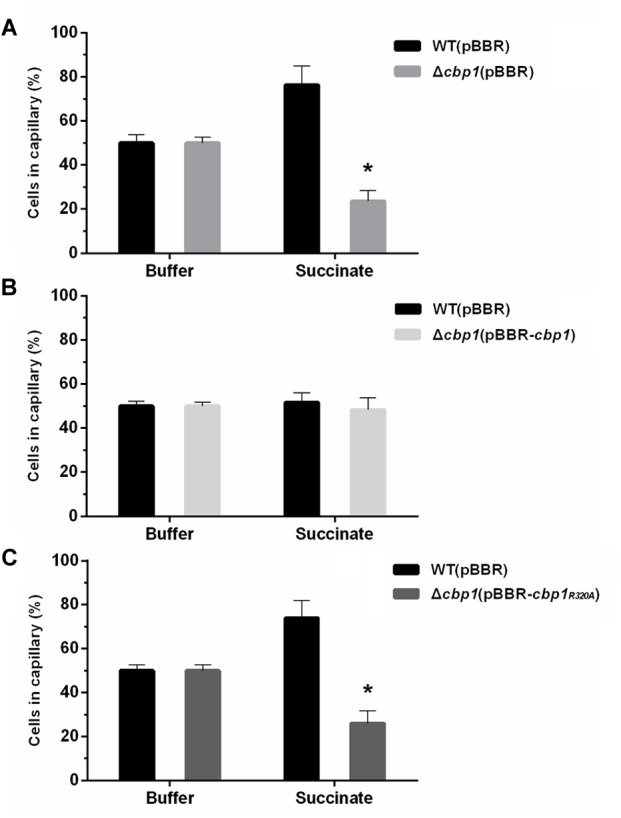
Chemotactic behavior of wild-type ORS571 and mutants. **(A)** Competitive chemotactic responses of wild type and Δ*cbp1* mutant using the quantitative capillary assay. **(B)** Competitive chemotactic responses of wild type and Δ*cbp1*(pBBR-*cbp1*) mutant using the quantitative capillary assay. **(C)** Competitive chemotactic responses of wild type and Δ*cbp1*(pBBR-*cbp1_R320A_*) mutant using the quantitative capillary assay. Wild type and each mutant were mixed in a 1:1 ratio. The capillary contained either PBS buffer as control or 10 mM succinate solution as a chemoattractant. Error bars indicate standard deviations for three independent biological replicates and asterisks represent significant differences (^∗^*P* < 0.01).

To determine the role of Cbp1 in chemotaxis, a Cbp1 deletion mutant (Δ*cbp1*) was constructed. The growth curves of the strains were first determined and the results suggested that the growth rate of the Δ*cbp1* mutant had no obvious difference compared with that of the wild type in both rich (TY) and minimal media (L3+N) with succinate as a carbon source (Supplementary Figure S2). The competitive capillary chemotaxis assay was performed to characterize the chemotactic behavior of wild type and mutants. For the competitive capillary assay, wild type and the Δ*cbp1* mutant were mixed in a 1:1 ratio and capillaries containing PBS buffer or the chemoattractant succinate were immersed in bacterial suspensions. As shown in Figure 3A, the number of Δ*cbp1* mutant cells entering the capillary containing PBS buffer was about the same as the wild type (50% vs. 50%), which suggested that the swimming ability of the Δ*cbp1* mutant was not affected. However, cells of the Δ*cbp1* mutant entering the capillary containing the chemoattractant succinate were greatly reduced compared to the wild type (23.5% vs. 76.5%), confirming that the chemotaxis ability of the Δ*cbp1* mutant was impaired.

Since Cbp1 binds c-di-GMP, the role of c-di-GMP in chemotaxis may be of great importance. To investigate any potential interaction between c-di-GMP and chemotaxis, genetic complementation experiments were performed using the Δ*cbp1* mutant and broad-host-range plasmids expressing wild-type *cbp1* or *cbp1_R320A_* under the control of their native promoters. As shown in Figure 3B, cells of the complemented strain Δ*cbp1* (pBBR*-cbp1*) entering the capillary containing chemoattractant were about the same as the wild type, suggesting that the chemotaxis defect of the Δ*cbp1* mutant was completely restored by the expression of wild-type *cbp1*. However, the plasmid pBBR*-cbp1_R320A_* failed to restore the chemotaxis defect of the Δ*cbp1* mutant (Figure 3C). The result suggested that loss of c-di-GMP binding ability significantly impaired chemotaxis and the c-di-GMP binding ability of Cbp1 was essential for normal chemotaxis.

### The Δ*cbp1* Mutant Has Enhanced Aggregation Ratio

**FIGURE 4:**
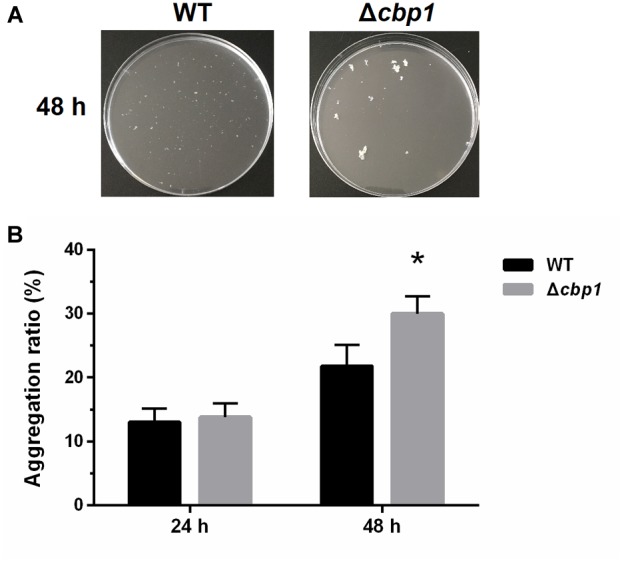
Aggregation of wild-type ORS571 and the Δ*cbp1* mutant. **(A)** The aggregation morphologies of wild type and Δ*cbp1* mutant in L3+N liquid medium after 48 h of incubation. **(B)** The aggregation ratios of wild type and Δ*cbp1* mutant. Error bars indicate standard deviations for three independent biological replicates and asterisk represent significant difference (^∗^*P* < 0.01).

As previously reported, *A. caulinodans* ORS571 can aggregate and form flocs in minimal medium when incubated with high rotational speed (Nakajima et al., 2012). Chemotaxis has been implicated in the regulation of cell aggregation (Alexandre, 2015). To determine whether Cbp1 played a role in aggregation, wild type and Δ*cbp1* mutant cells were analyzed for their ability to aggregate. As shown in Figure 4, the aggregation ratios of the wild type and Δ*cbp1* mutant were similar after 24 h of incubation. Aggregation and floc formation enhanced over time for both strains. However, after 48 h of incubation, the Δ*cbp1* mutant showed elevated aggregation ratio compared with the wild type (31% vs. 22%) and the flocs formed by the Δ*cbp1* mutant were also bigger than those of the wild type.

### The Δ*cbp1* Mutant Has Enhanced Biofilm Formation

**FIGURE 5:**
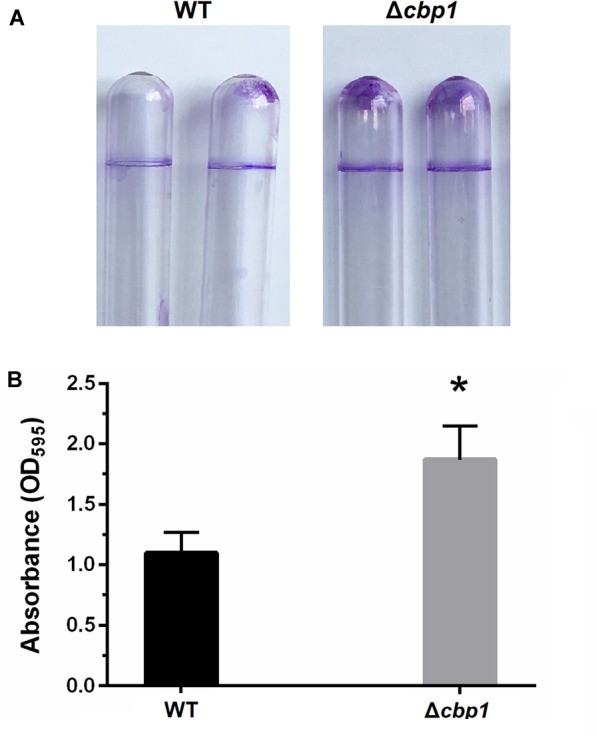
Biofilm formation of wild-type ORS571 and the Δ*cbp1* mutant. **(A)** The biofilm morphologies of wild type and Δ*cbp1* mutant following crystal violet staining. **(B)** Quantification of crystal violet-stained biofilms. Error bars indicate standard deviations for three independent biological replicates and asterisk represent significant difference (^∗^*P* < 0.01).

Besides cell aggregation, chemotaxis also has a pronounced effect on biofilm formation under static conditions (Liu et al., 2018). Thus, biofilm formation of the Δ*cbp1* mutant on abiotic surfaces was measured using crystal violet staining. After 3 days of incubation, the biofilm formed at the air-liquid interface by the Δ*cbp1* mutant appeared to be thicker than that of the wild type (Figure 5A), which was confirmed by quantitative determination (Figure 5B). This indicates that the chemoreceptor Cbp1 may modulate biofilm formation.

### The Δ*cbp1* Mutant Is Impaired in Competitive Root Colonization and Nodulation

**FIGURE 6:**
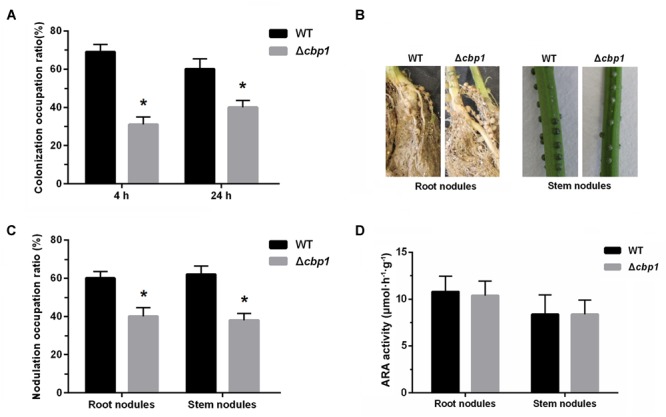
Root colonization, nodulation, and nitrogen fixation of wild-type ORS571 and the Δ*cbp1* mutant. **(A)** The competitive root colonization ratio of wild type and Δ*cbp1* mutant. **(B)** The morphologies of root nodules and stem nodules induced by wild type and Δ*cbp1* mutant. **(C)** The competitive root and stem nodulation ratio of wild type and Δ*cbp1* mutant. **(D)** The nitrogen fixation efficiencies of wild type and Δ*cbp1* mutant. Error bars indicate standard deviations for three independent biological replicates and asterisks represent significant differences (^∗^*P* < 0.01).

Establishment of the rhizobia-legume symbiosis depends on colonization of the root system, which itself involves bacterial chemotaxis toward roots (Greer-Phillips et al., 2004). Since the Δ*cbp1* mutant was impaired in chemotaxis, we expected that it would also be impaired in root colonization. Colonization abilities of both strains on the root surface were assessed. Seedlings were placed in vermiculite containing a mixture of both strains (1:1 ratio) for 4 and 24 h. The colonized strains were then re-isolated and quantified. As shown in Figure 6A, the wild type performed better than the Δ*cbp1* mutant (69% of wild type vs. 31% of Δ*cbp1* mutant) in root colonization after 4 h of incubation. The result suggested that the Δ*cbp1* mutant was less competitive than the wild type in root surface colonization and the defect in chemotaxis affected the initial colonization of rhizobia on legume roots. After 24 h of incubation, the occupation ratio of the wild type and the Δ*cbp1* mutant was 60%: 40%. The slightly elevated occupation ratio of the Δ*cbp1* mutant may suggest that the lower colonization efficiency caused by the chemotaxis defect could be partially compensated with a longer incubation time.

Nodulation of wild type and the Δ*cbp1* mutant were then further tested. Figure 6B shows that, similar to the wild type, the Δ*cbp1* mutant successfully induced nodules on both roots and stems. However, when competitive experiments were performed, using wild type and the Δ*cbp1* mutant at a 1: 1 ratio, the Δ*cbp1* mutant was impaired in competitive nodulation on both roots and stems. The number of root nodules formed by the wild type was about 1.5-fold higher than that of the Δ*cbp1* mutant and the ratio for stem nodules was about the same (Figure 6C). To test whether the Δ*cbp1* mutant induced functional nodules, the ARA of nodules was also determined. The results showed that the ARA of nodules formed by the Δ*cbp1* mutant was similar as that of wild type (Figure 6D). Together, these data suggested that Cbp1 was involved in competitive colonization and nodulation, but the nitrogen fixation of ORS571 was not affected by a lack of Cbp1.

## Discussion

In this study, the chemoreceptor Cbp1 was shown to bind c-di-GMP and a mutation in the conserved RxxxR motif caused complete loss of c-di-GMP binding. In other systems, the role of the five key residues of the RxxxR and D/NxSxxG motifs of the PilZ domain in c-di-GMP binding has been extensively analyzed. For YcgR from *E. coli*, substitution of second Arg (from the RxxxR motif) with Asp completely abolished c-di-GMP binding, while the role of Ser residue (from the D/NxSxxG) in c-di-GMP binding was not critical (Ryjenkov et al., 2006). For DgrA from *Caulobacter crescentus*, the first Arg residue of the RxxxR motif was shown to be essential for c-di-GMP binding (Christen et al., 2007). In this study, replacement of the first Arg residue of the RxxxR motif by Ala also resulted in the complete loss of c-di-GMP binding ability of Cbp1. Thus, we have confirmed the essential role of the RxxxR motif in c-di-GMP binding.

In this study, we have determined that Cbp1 binds c-di-GMP with a *K*_D_ of 14.94 ± 1.6 μM. C-di-GMP binding affinities of PilZ domain proteins span a sizable range of c-di-GMP concentrations. In *Salmonella typhimurium*, the *K*_D_ of YcgR for c-di-GMP is 0.19 ± 0.018 μM, while BcsA binds c-di-GMP with a *K*_D_ of 8.24 ± 2.4 μM (Pultz et al., 2012). In *P. aeruginosa*, MapZ and Alg44 bind c-di-GMP with *K*_D_ of 8.8 ± 1.2 and 12.74 ± 1.68 μM, respectively (Pultz et al., 2012; Xu et al., 2016b). The PilZ domain containing chemoreceptor Tlp1 from *A. brasiencse* binds c-di-GMP with a moderate *K*_D_ of 6.9 ± 1.2 μM (Russell et al., 2013). The intracellular concentration of c-di-GMP is speculated to be in the submicromolar to low micromolar range, but local concentrations may be considerably higher (Hengge, 2009). The range of c-di-GMP binding affinities of PilZ domain proteins correlates well with that of intracellular c-di-GMP concentrations.

There are 43 chemoreceptors and a single chemotaxis pathway (CheAWY_1_BR) in *A. caulinodans* ORS571 (Jiang et al., 2016; Liu et al., 2018). Chemoreceptors are responsible for sensing signals that are then transmitted to the coupling protein CheW and the histidine kinase CheA. Next, CheY, the response regulator of CheA, regulates the directional change of flagellar rotation (Wuichet et al., 2007). Intracellular concentrations of c-di-GMP respond to a variety of external signals (Schirmer, 2016). As a c-di-GMP binding chemoreceptor, Cbp1 may be responsible for sensing changes in intracellular c-di-GMP concentrations and transmitting the signal to downstream chemotaxis proteins. It is known that chemoreceptor interacts with downstream CheA/CheW, and the initiation of signal transduction is the conformational change of chemoreceptor upon ligand binding (Lai et al., 2006). The result of thermal shift assay showed that binding of c-di-GMP caused significant conformational change of Cbp1. The chemotaxis defect caused by lacking of c-di-GMP binding ability of Cbp1 suggested that the c-di-GMP-induced conformational change may be essential for Cbp1-mediated chemotaxis.

As adaptation proteins present in the chemotaxis pathway, methylesterase CheB and methyltransferase CheR compete with each other to regulate methylation state and ligand binding affinity of chemoreceptors to reset chemotaxis sensitivity (Li and Hazelbauer, 2005). The PilZ domain protein MapZ from *P. aeruginosa* has been shown to interact with CheR1 (Xu et al., 2016b). C-di-GMP binding to MapZ induces dramatic conformational changes in MapZ. The conformational changes are crucial for MapZ-CheR1 binding, which regulates the activity of CheR1 and the methylation state of chemoreceptor PctA (Yan et al., 2018). In ORS571, there is another possibility that Cbp1 interacts with the adaptation protein CheR via its PilZ domain at the presence of c-di-GMP. The potential interaction between Cbp1 and CheR may regulate the activity of CheR and chemotaxis sensitivity. The impaired chemotaxis of the Δ*cbp1* mutant may result from lacking of functional adaptational modification of other chemoreceptors. However, the underlying mechanistic function of Cbp1 remains to be further investigated. An analysis of 20681 chemoreceptors has shown that 52 of them contained a C-terminal PilZ domain, which suggests that the integration of c-di-GMP into chemotaxis is not unique to only one or two species (Russell et al., 2013).

Chemotaxis allows motile bacteria to rapidly move toward beneficial niches or away from detrimental conditions, thus affecting the transient cell-cell contact and the probability of cell clumping, which leads to aggregation (Alexandre, 2015). It has been reported that *A. brasilense* mutants with defective chemotaxis ability had elevated aggregation ratios compared with wild type (Bible et al., 2012). In this study, the Δ*cbp1* mutant aggregated more than the wild-type ORS571 after long incubation. The defect in chemotaxis caused by loss of the chemoreceptor Cbp1 may account for the enhanced aggregation of the Δ*cbp1* mutant. Besides cell-cell contact, chemotaxis also affects cell-surface interactions involved in biofilm formation. PilZ domain containing proteins have been implicated in the regulation of biofilm formation (Martinez-Granero et al., 2014). Because of the dual role of Cbp1 in chemotaxis and the c-di-GMP signal transduction network, biofilm formation by the Δ*cbp1* mutant may be regulated by these two mechanisms together.

Chemotaxis has been shown to play an important role in root surface colonization and nodulation. For example, *Pseudomonas putida* KT2440 colonized corn root surface by rapid chemotaxis, chemotaxis enhanced the root colonization of the PGPR (plant-growth-promoting rhizobacteria) strain *Bacillus amyloliquefaciens* SQR9 on cucumber roots, and the *che1* chemotaxis gene cluster of *Rhizobium leguminosarum* was found to be essential for competitive nodulation (Espinosa-Urgel et al., 2002; Miller et al., 2007; Feng et al., 2018). As previously reported, an *A. brasilense* Sp7 mutant lacking the energy-sensing chemoreceptor Tlp1 was severely impaired in chemotaxis and root surface colonization of wheat (Greer-Phillips et al., 2004). Another study in our lab showed that an *A. caulinodans* ORS571 mutant Δ*tlpA1* with a chemotaxis defect was also compromised in competitive root surface colonization and nodulation of *S. rostrata* (Liu et al., 2017). Consistent with the former findings, the competitive root colonization efficiency of the Δ*cbp1* mutant was lower than that of the wild-type ORS571. The results further showed the vital role of chemotaxis in competitive colonization and nodulation.

## Conclusion

In conclusion, we identified a c-di-GMP binding chemoreceptor Cbp1 in *A. caulinodans* ORS571, showing that the Δ*cbp1* mutant was impaired in chemotaxis and competitive root colonization and nodulation of the host legume *S. rostrata*. Our research provides an example of the interplay between chemotaxis and c-di-GMP, which may benefit our understanding of bacterial signal transduction networks.

## Data Availability

All datasets generated for this study are included in the manuscript and/or the Supplementary Files.

## Author Contributions

YS and ZX conceived and designed the experiments and wrote the manuscript. YS, FS, and XL carried out the experiments. YS analyzed data and prepared figures and tables. WC helped with the improvement and revision of the manuscript. All authors approved the submission for publication.

## Conflict of Interest Statement

WC was employed by the company Shandong Huibang Bohai Agriculture Development Limited Company. The remaining authors declare that the research was conducted in the absence of any commercial or financial relationships that could be construed as a potential conflict of interest.

## References

[B1] Alexandre G. (2015). Chemotaxis control of transient cell aggregation. *J. Bacteriol.* 197 3230–3237. 10.1128/JB.00121-15 26216846 PMC4573731

[B2] Benach J. Swaminathan S. Tamayo R. Handelman S. Folta-Stogniew E. Ramos J. (2007). The structural basis of cyclic diguanylate signal transduction by PilZ domains. *EMBO J.* 26 5153–5166. 10.1038/sj.emboj.7601918 18034161 PMC2140105

[B3] Bible A. Russell M. H. Alexandre G. (2012). The *Azospirillum brasilense* Che1 chemotaxis pathway controls swimming velocity, which affects transient cell-to-cell clumping. *J. Bacteriol.* 194 3343–3355. 10.1128/JB.00310-12 22522896 PMC3434747

[B4] Bordeleau E. Purcell E. B. Lafontaine D. A. Fortier L. C. Tamayo R. Burrus V. (2015). Cyclic di-GMP riboswitch-regulated type IV pili contribute to aggregation of *Clostridium difficile*. *J. Bacteriol.* 197 819–832. 10.1128/JB.02340-14 25512308 PMC4325102

[B5] Chan C. Paul R. Samoray D. Amiot N. C. Giese B. Jenal U. (2004). Structural basis of activity and allosteric control of diguanylate cyclase. *Proc. Natl. Acad. Sci. U.S.A.* 101 17084–17089. 10.1073/pnas.0406134101 15569936 PMC535365

[B6] Christen M. Christen B. Allan M. G. Folcher M. Jeno P. Grzesiek S. (2007). DgrA is a member of a new family of cyclic diguanosine monophosphate receptors and controls flagellar motor function in *Caulobacter crescentus*. *Proc. Natl. Acad. Sci. U.S.A.* 104 4112–4117. 10.1073/pnas.0607738104 17360486 PMC1805490

[B7] Christen M. Christen B. Folcher M. Schauerte A. Jenal U. (2005). Identification and characterization of a cyclic di-GMP-specific phosphodiesterase and its allosteric control by GTP. *J. Biol. Chem.* 280 30829–30837. 10.1074/jbc.M504429200 15994307

[B8] Dreyfus B. Garcia J. L. Gillis M. (1988). Characterization of *Azorhizobium caulinodans* gen. nov., sp. nov., a stem nodulating nitrogen-fixing bacterium isolated from *Sesbania rostrata*. *Int. J. Syst. Bacteriol.* 38 89–98. 10.1099/00207713-38-1-89

[B9] Espinosa-Urgel M. Kolter R. Ramos J. (2002). Root colonization by *Pseudomonas putida*: love at first sight. *Microbiology* 148 341–343. 10.1099/00221287-148-2-341 11832496

[B10] Feng H. Zhang N. Du W. Zhang H. Liu Y. Fu R. (2018). Identification of chemotaxis compounds in root exudates and their sensing chemoreceptors in plant-growth-promoting rhizobacteria *Bacillus amyloliquefaciens* SQR9. *Mol. Plant Microbe Interact.* 31 995–1005. 10.1094/MPMI-01-18-0003-R 29714096

[B11] Figurski D. H. Helinski D. R. (1979). Replication of an origin-containing derivative of plasmid RK2 dependent on a plasmid function provided in trans. *Proc. Natl. Acad. Sci. U.S.A.* 76 1648–1652. 10.1073/pnas.76.4.1648 377280 PMC383447

[B12] Gao S. Romdhane S. B. Beullens S. Kaever V. Lambrichts I. Fauvart M. (2014). Genomic analysis of cyclic-di-GMP-related genes in rhizobial type strains and functional analysis in *Rhizobium etli*. *Appl. Microbiol. Biotechnol.* 98 4589–4602. 10.1007/s00253-014-5722-7 24728599

[B13] Greer-Phillips S. E. Stephens B. B. Alexandre G. (2004). An energy taxis transducer promotes root colonization by *Azospirillum brasilense*. *J. Bacteriol.* 186 6595–6604. 10.1128/JB.186.19.6595-6604.2004 15375141 PMC516605

[B14] Hengge R. (2009). Principles of c-di-GMP signalling in bacteria. *Nat. Rev. Microbiol.* 7 263–273. 10.1038/nrmicro2109 19287449

[B15] Huang Z. Ni B. Jiang C. Y. Wu Y. F. He Y. Z. Parales R. E. (2016). Direct sensing and signal transduction during bacterial chemotaxis toward aromatic compounds in *Comamonas testosteroni*. *Mol. Microbiol.* 101 224–237. 10.1111/mmi.13385 27008921

[B16] Huynh K. Partch C. L. (2015). Analysis of protein stability and ligand interactions by thermal shift assay. *Curr. Protoc. Protein Sci.* 79 28.9.1–28.9.14. 10.1002/0471140864.ps2809s79 25640896 PMC4332540

[B17] Jiang N. Liu W. Li Y. Wu H. Zhang Z. Alexandre G. (2016). A chemotaxis receptor modulates nodulation during the *Azorhizobium caulinodans-Sesbania rostrata* symbiosis. *Appl. Environ. Microbiol.* 82 3174–3184. 10.1128/AEM.00230-16 26994081 PMC4959239

[B18] Kovach M. Elzer P. Hill D. Robertson G. Farris M. Martin Roop R. II (1995). Four new derivatives of the broad-host-range cloning vector pBBR1MCS, carrying different antibiotic-resistance cassettes. *Gene* 166 175–176. 10.1016/0378-1119(95)00584-1 8529885

[B19] Lai W. C. Beel B. D. Hazelbauer G. L. (2006). Adaptational modification and ligand occupancy have oppsite effects on positioning of the transmembrane signalling helix of a chemoreceptor. *Mol. Microbiol.* 61 1081–1090. 10.1111/j.1365-2958.2006.05296.x 16879656

[B20] Lee K. B. De Backer P. Aono T. Liu C. T. Suzuki S. Suzuki T. (2008). The genome of the versatile nitrogen fixer *Azorhizobium caulinodans* ORS571. *BMC Genomics* 9:271. 10.1186/1471-2164-9-271 18522759 PMC2443382

[B21] Lee V. T. Matewish J. M. Kessler J. L. Hyodo M. Hayakawa Y. Lory S. (2007). A cyclic-di-GMP receptor required for bacterial exopolysaccharide production. *Mol. Microbiol.* 65 1474–1484. 10.1111/j.1365-2958.2007.05879.x 17824927 PMC2170427

[B22] Li G. Weis R. M. (2000). Covalent modification regulates ligand binding to receptor complexes in the chemosensory system of *Escherichia coli*. *Cell* 100 357–365. 10.1016/S0092-8674(00)80671-6 10676817

[B23] Li M. Hazelbauer G. L. (2005). Adaptational assistance in clusters of bacterial chemoreceptors. *Mol. Microbiol.* 56 1617–1626. 10.1111/j.1365-2958.2005.04641.x 15916610

[B24] Liu W. Sun Y. Shen R. Dang X. Liu X. Sui F. (2018). A chemotaxis-like pathway of *Azorhizobium caulinodans* controls flagella-driven motility, which regulates biofilm formation, exopolysaccharide biosynthesis, and competitive nodulation. *Mol. Plant Microbe Interact.* 31 737–749. 10.1094/MPMI-12-17-0290-R 29424664

[B25] Liu W. Yang J. Sun Y. Liu X. Li Y. Zhang Z. (2017). *Azorhizobium caulinodans* transmembrane chemoreceptor TlpA1 involved in host colonization and nodulation on roots and stems. *Front. Microbiol.* 8:1327. 10.3389/fmicb.2017.01327 28751887 PMC5508009

[B26] Martinez-Granero F. Navazo A. Barahona E. Redondo-Nieto M. Gonzalez de Heredia E. Baena I. (2014). Identification of flgZ as a flagellar gene encoding a PilZ domain protein that regulates swimming motility and biofilm formation in *Pseudomonas*. *PLoS One* 9:e87608. 10.1371/journal.pone.0087608 24504373 PMC3913639

[B27] Marx C. J. Lidstrom M. E. (2002). Broad-host-range *cre-lox* system for antibiotic marker recycling in Gram-negative bacteria. *Biotechniques* 22 1062–1067. 10.2144/02335rr01 12449384

[B28] Merighi M. Lee V. T. Hyodo M. Hayakawa Y. Lory S. (2007). The second messenger bis-(3′-5′)-cyclic-GMP and its PilZ domain-containing receptor Alg44 are required for alginate biosynthesis in *Pseudomonas aeruginosa*. *Mol. Microbiol.* 65 876–895. 10.1111/j.1365-2958.2007.05817.x 17645452

[B29] Miller L. D. Yost C. K. Hynes M. F. Alexandre G. (2007). The major chemotaxis gene cluster of *Rhizobium leguminosarum* bv. viciae is essential for competitive nodulation. *Mol. Microbiol.* 63 348–362. 10.1111/j.1365-2958.2006.05515.x 17163982

[B30] Nakajima A. Aono T. Tsukada S. Siarot L. Ogawa T. Oyaizu H. (2012). Lon protease of *Azorhizobium caulinodans* ORS571 is required for suppression of *reb* gene expression. *Appl. Environ. Microbiol.* 78 6251–6261. 10.1128/AEM.01039-12 22752172 PMC3416630

[B31] Newell P. D. Monds R. D. O’Toole G. A. (2009). LapD is a bis-(3′,5′)-cyclic dimeric GMP-binding protein that regulates surface attachment by *Pseudomonas fluorescens* Pf0-1. *Proc. Natl. Acad. Sci. U.S.A.* 106 3461–3466. 10.1073/pnas.0808933106 19218451 PMC2651287

[B32] Paul K. Nieto V. Carlquist W. C. Blair D. F. Harshey R. M. (2010). The c-di-GMP binding protein YcgR controls flagellar motor direction and speed to affect chemotaxis by a “backstop brake” mechanism. *Mol. Cell* 38 128–139. 10.1016/j.molcel.2010.03.001 20346719 PMC2929022

[B33] Povolotsky T. L. Hengge R. (2012). ‘Life-style’ control networks in *Escherichia coli*: signaling by the second messenger c-di-GMP. *J. Biotechnol.* 160 10–16. 10.1016/j.jbiotec.2011.12.024 22226726

[B34] Pultz I. S. Christen M. Kulasekara H. D. Kennard A. Kulasekara B. Miller S. I. (2012). The response threshold of *Salmonella* PilZ domain proteins is determined by their binding affinities for c-di-GMP. *Mol. Microbiol.* 86 1424–1440. 10.1111/mmi.12066 23163901 PMC5034864

[B35] Purcell E. B. McKee R. W. McBride S. M. Waters C. M. Tamayo R. (2012). Cyclic diguanylate inversely regulates motility and aggregation in *Clostridium difficile*. *J. Bacteriol.* 194 3307–3316. 10.1128/JB.00100-12 22522894 PMC3434733

[B36] Rao F. Yang Y. Qi Y. Liang Z. X. (2008). Catalytic mechanism of cyclic di-GMP-specific phosphodiesterase: a study of the EAL domain-containing RocR from *Pseudomonas aeruginosa*. *J. Bacteriol.* 190 3622–3631. 10.1128/JB.00165-08 18344366 PMC2394985

[B37] Roy A. B. Petrova O. E. Sauer K. (2012). The phosphodiesterase DipA (PA5017) is essential for *Pseudomonas aeruginosa* biofilm dispersion. *J. Bacteriol.* 194 2904–2915. 10.1128/JB.05346-11 22493016 PMC3370607

[B38] Russell M. H. Bible A. N. Fang X. Gooding J. R. Campagna S. R. Gomelsky M. (2013). Integration of the second messenger c-di-GMP into the chemotactic signaling pathway. *mBio* 4:e00001-13. 10.1128/mBio.00001-13 23512960 PMC3604760

[B39] Ryan R. P. Fouhy Y. Lucey J. F. Crossman L. C. Spiro S. He Y. W. (2006). Cell-cell signaling in *Xanthomonas campestris* involves an HD-GYP domain protein that functions in cyclic di-GMP turnover. *Proc. Natl. Acad. Sci. U.S.A.* 103 6712–6717. 10.1073/pnas.0600345103 16611728 PMC1458946

[B40] Ryan R. P. Tolker-Nielsen T. Dow J. M. (2012). When the PilZ don’t work: effectors for cyclic di-GMP action in bacteria. *Trends Microbiol.* 20 235–242. 10.1016/j.tim.2012.02.008 22444828

[B41] Ryjenkov D. A. Simm R. Romling U. Gomelsky M. (2006). The PilZ domain is a receptor for the second messenger c-di-GMP: the PilZ domain protein YcgR controls motility in enterobacteria. *J. Biol. Chem.* 281 30310–30314. 10.1074/jbc.C600179200 16920715

[B42] Ryjenkov D. A. Tarutina M. Moskvin O. V. Gomelsky M. (2005). Cyclic diguanylate is a ubiquitous signaling molecule in bacteria: insights into biochemistry of the GGDEF protein domain. *J. Bacteriol.* 187 1792–1798. 10.1128/JB.187.5.1792-1798.2005 15716451 PMC1064016

[B43] Schäper S. Krol E. Skotnicka D. Kaever V. Hilker R. Sogaard-Andersen L. (2016). Cyclic di-GMP regulates multiple cellular functions in the symbiotic alphaproteobacterium *Sinorhizobium meliloti*. *J. Bacteriol.* 198 521–535. 10.1128/JB.00795-15 26574513 PMC4719442

[B44] Scharf B. E. Hynes M. F. Alexandre G. M. (2016). Chemotaxis signaling systems in model beneficial plant-bacteria associations. *Plant Mol. Biol.* 90 549–559. 10.1007/s11103-016-0432-4 26797793

[B45] Schirmer T. (2016). C-di-GMP Synthesis: structural aspects of evolution, catalysis and regulation. *J. Mol. Biol.* 428 3683–3701. 10.1016/j.jmb.2016.07.023 27498163

[B46] Sun Y. Zhao H. Wang J. Zhu J. Wu S. (2015). Identification and regulation of the catalytic promiscuity of (-)-gamma-lactamase from *Microbacterium hydrocarbonoxydans*. *Appl. Microbiol. Biotechnol.* 99 7559–7568. 10.1007/s00253-015-6503-7 25773976

[B47] Tindall M. J. Gaffney E. A. Maini P. K. Armitage J. P. (2012). Theoretical insights into bacterial chemotaxis. *Wiley Interdiscip. Rev. Syst. Biol. Med.* 4 247–259. 10.1002/wsbm.1168 22411503

[B48] Wadhams G. H. Armitage J. P. (2004). Making sense of it all: bacterial chemotaxis. *Nat. Rev. Mol. Cell Biol.* 5 1024–1037. 10.1038/nrm1524 15573139

[B49] Wuichet K. Alexander R. P. Zhulin I. B. (2007). Comparative genomic and protein sequence analyses of a complex system controlling bacterial chemotaxis. *Methods Enzymol.* 422 3–31. 10.1016/S0076-6879(06)22001-9 17628132 PMC2754700

[B50] Xu L. Venkataramani P. Ding Y. Liu Y. Deng Y. Yong G. L. (2016a). A cyclic di-GMP-binding adaptor protein interacts with histidine kinase to regulate two-component signaling. *J. Biol. Chem.* 291 16112–16123. 10.1074/jbc.M116.730887 27231351 PMC4965561

[B51] Xu L. Xin L. Zeng Y. Yam J. Ding Y. Venkataramani P. (2016b). A cyclic di-GMP-binding adaptor protein interacts with a chemotaxis methyltransferase to control flagellar motor switching. *Sci. Signal.* 9:ra102. 27811183 10.1126/scisignal.aaf7584

[B52] Yan X. F. Xin L. Yen J. T. Zeng Y. Jin S. Cheang Q. W. (2018). Structural analyses unravel the molecular mechanism of cyclic di-GMP regulation of bacterial chemotaxis via a PilZ adaptor protein. *J. Biol. Chem.* 293 100–111. 10.1074/jbc.M117.815704 29146598 PMC5766925

[B53] Yang F. Tian F. Chen H. Hutchins W. Yang C. H. He C. (2015). The *Xanthomonas oryzae* pv. oryzae PilZ domain proteins function differentially in cyclic di-GMP binding and regulation of virulence and motility. *Appl. Environ. Microbiol.* 81 4358–4367. 10.1128/AEM.04044-14 25911481 PMC4475898

