# Supplementary material for: Identification of Cbp1, a c-di-GMP Binding Chemoreceptor in Azorhizobium caulinodans ORS571 Involved in Chemotaxis and Nodulation of the Host Plant
Source: Front Microbiol. 2019 Apr 2;10:638. doi: 10.3389/fmicb.2019.00638 (PMC6454048; doi:10.3389/fmicb.2019.00638)

Identification of Cbp1, a c-di-GMP binding chemoreceptor in *Azorhizobium caulinodans* ORS571 involved in chemotaxis and nodulation of the host plant.

Yu Sun[1, 2] , Zhihong Xie[1*], Fu Sui[1, 2] , Wei liu[1] , Xiaolin Liu[1, 2]

1. Key Laboratory of Coastal Biology and Bioresource Utilization, Yantai Institute of Coastal Zone Research, Chinese Academy of Sciences, Yantai, China;
2. University of Chinese Academy of Sciences, Beijing, China.

Running title:
Identification of c-di-GMP binding chemoreceptor

Correspondence:
Dr. Zhihong Xie

zhxie@yic.ac.cn

2 figures

Figure S1. Cbp1 representation, purification, and multiple sequence alignment. (A) Illustration of the 4,000 bp DNA region encompassing the *cbp1* gene (*AZC_3349*) and the domain architecture predicted by Pfam. The direction of transcription is indicated by the arrow. MA domain: <u>m</u>ethyl-<u>a</u>ccepting chemotaxis-like domain (B) SDS-PAGE analysis of purified Cbp1 and Cbp1R320A proteins. Cbp1 protein with C-His6 tag is 64.49 kDa. (C) Multiple alignment of protein sequences of the PilZ domain-containing proteins of A*zorhizobium caulinodans* ORS571 and other strains using CLUSTAL_W. Each sequence in the alignment is identified by its GI (<u>G</u>enBank protein <u>I</u>dentification) number. Abbreviations: Acau, *Azorhizobium caulinodans* ORS571; Abra, *Azospirillum brasilense* Sp7; Smel, *Sinorhizobium meliloti* 1021; Ecol, *Escherichia coli* K-12; Paer, *Pseudomonas aeruginosa* PAO1; Xory; *Xanthomonas oryzae* pv. oryzae. The asterisks (*) indicate the conserved RxxxR and D/NxSxxG motifs, and the black triangle (▲) indicates the key residue Arg320. Conserved residues are highlighted in black and grey according to their degree of conservation.

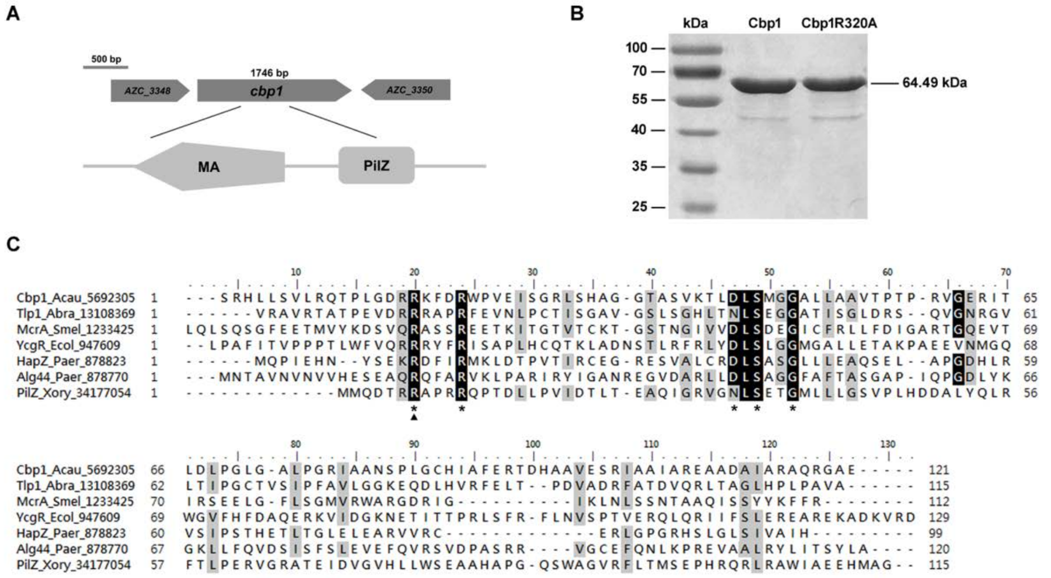

Figure S2. Growth curves of wild-type ORS571 and mutants. Growth curves of wild type, Δ*cbp1* mutant, Δ*cbp1*(pBBR-*cbp1*) mutant, and Δ*cbp1*(pBBR-$cbp1_{R320A}$) mutant in L3+N medium with succinate as a carbon source (A) and TY medium (B). Cells were grown at 150 rpm and 37℃ and cell density was monitored every 2 h (0-12 h), 3 h (12-24 h) and 6 h (24-48 h). The results represent the average of three independent biological replicates.

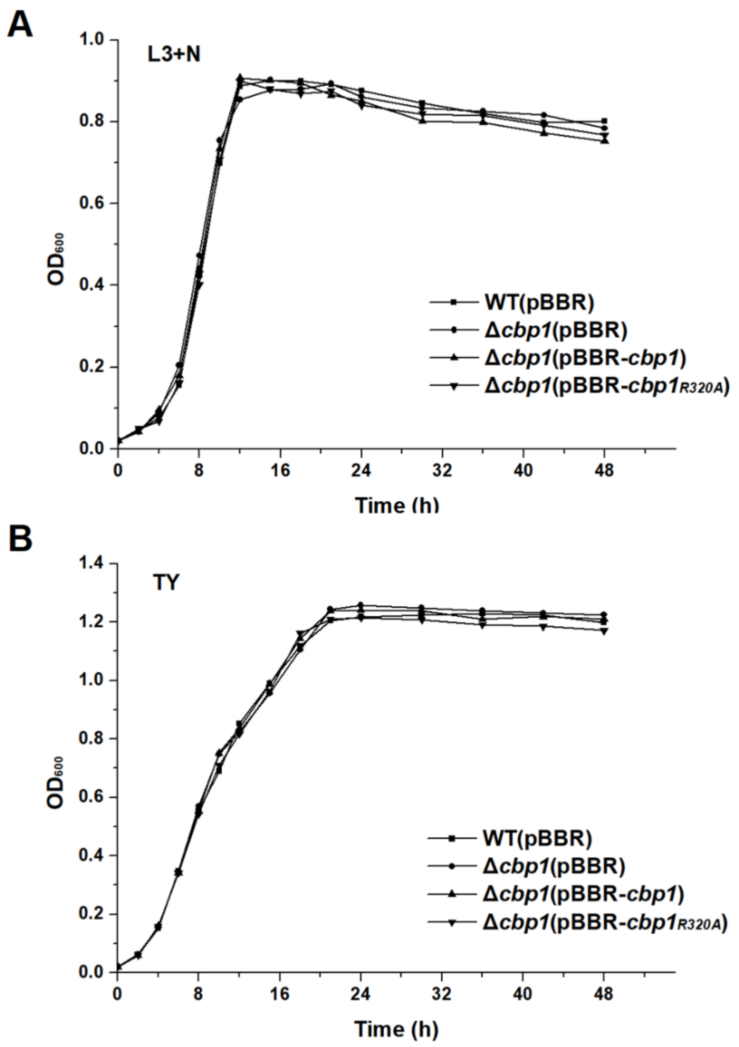

Supplement: Supplementary file 1 [file Data_Sheet_1.PDF]
